# Assessing and Improving the Care of Patients With Heart Failure in Ghana: Protocol for a Prospective Observational Study and the Ghana Heart Initiative-Heart Failure Registry

**DOI:** 10.2196/52616

**Published:** 2024-04-08

**Authors:** Felix Awindaogo, Emmanuel Acheamfour-Akowuah, Alfred Doku, Collins Kokuro, Francis Agyekum, Isaac Kofi Owusu

**Affiliations:** 1 Korle-Bu Teaching Hospital Accra Ghana; 2 Directorate of Medicine Kumasi Komfo Anokye Teaching Hospital Kumasi Ghana; 3 Department of Medicine and Therapeutics University of Ghana Medical School University of Ghana Accra Ghana; 4 Department of Medicine School of Medicine and Dentistry Kwame Nkrumah University of Science and Technology Kumasi Ghana

**Keywords:** clinical, cross-sectional, epidemiology, Ghana, heart failure, heart, management, medium-term, monitoring, mortality, outcome, patient data, prevention, protocol, teaching, treatment

## Abstract

**Background:**

Heart failure (HF) is a leading cause of morbidity and mortality globally, with a high disease burden. The prevalence of HF in Ghana is increasing rapidly, but epidemiological profiles, treatment patterns, and survival data are scarce. The national capacity to diagnose and manage HF appropriately is also limited. To address the growing epidemic of HF, it is crucial to recognize the epidemiological characteristics and medium-term outcomes of HF in Ghana and improve the capability to identify and manage HF promptly and effectively at all levels of care.

**Objective:**

This study aims to determine the epidemiological characteristics and medium-term HF outcomes in Ghana.

**Methods:**

We conducted a prospective, multicenter, multilevel cross-sectional observational study of patients with HF from January to December 2023. Approximately 5000 patients presenting with HF to 9 hospitals, including teaching, regional, and municipal hospitals, will be recruited and evaluated according to a standardized protocol, including the use of an echocardiogram and an N-terminal pro-brain natriuretic peptide (NT-proBNP) test. Guideline-directed medical treatment of HF will be initiated for 6 months, and the medium-term outcomes of interventions, including rehospitalization and mortality, will be assessed. Patient data will be collated into a HF registry for continuous assessment and monitoring.

**Results:**

This intervention will generate the necessary information on the etiology of HF, clinical presentations, the diagnostic yield of various tools, and management outcomes. In addition, it will build the necessary capacity and support for HF management in Ghana. As of July 30, 2023, the training and onboarding of all 9 centers had been completed. Preliminary analyses will be conducted by the end of the second quarter of 2024, and results are expected to be publicly available by the middle of 2024.

**Conclusions:**

This study will provide the necessary data on HF, which will inform decisions on the prevention and management of HF and form the basis for future research.

**Trial Registration:**

ISRCTN Registry (United Kingdom) ISRCTN18216214; https:www.isrctn.com/ISRCTN18216214

**International Registered Report Identifier (IRRID):**

DERR1-10.2196/52616

## Introduction

### Overview

Heart failure (HF) is a costly, multifaceted, and life-threatening syndrome characterized by significant morbidity and mortality. Globally, HF affects 64 million people, with a prevalence of 1%-2% of adults in the general population and an estimated incidence of 1-20 cases per 1000 person-years [1].

Sub-Saharan Africa (SSA) has no population-based data; however, in-hospital prevalence ranges from 9.4% to 42.5% [2,3]. HF in SSA mainly affects young people and middle-aged individuals, occurring in people aged between 36 and 62.4 years [4]. It poses a substantial disease burden, with high mortality, rehospitalization rates, and health care costs, primarily attributable to readmissions and prolonged hospitalization periods of 11-13 days [3,5,6].

HF contributes significantly to Ghana’s cardiovascular disease burden, with a worse prognosis and a more malignant course [7,8]. It is a leading cause of death among Ghanaian adults; yet, there is a paucity of data on the epidemiological profiles, treatment patterns, and survival rates of patients with HF in Ghana [9-11]. Single-center studies indicate a high prevalence of HF in Ghana [7,8,12].

The diagnosis of HF in most patients is primarily based on clinical manifestations due to the limited availability of diagnostic equipment. Ghana has few cardiologists, who are mainly located in tertiary hospitals [13]. In addition, there is a lack of HF education and training for physicians and nonphysician health workers. While HF management teams or multidisciplinary teams for HF management are the gold standard model for the delivery of care, these teams are nonexistent in Ghana, and most health facilities lack resources for long-term patient follow-up, such as diagnostic equipment, dedicated HF clinics, and protocols [14-17].

A national network of heart failure management teams (NNHFMT) will be established as part of the Ghana Heart Initiative’s efforts to improve cardiovascular disease care in Ghana to help mitigate the burden of HF. The NNHFMT is tasked with building the capacity of both secondary and tertiary levels of care to promptly and effectively identify and manage HF by creating heart failure management teams (HFMTs) and establishing a national registry for HF and HF clinics that will be integrated with routine clinical services to provide long-term follow-up and care. The establishment of HF clinics and a national registry will fill a significant gap in HF care and research by providing the most recent epidemiological, management patterns, and medium-term outcomes data on HF.

### Objectives

The primary objective of this study is to determine the epidemiological characteristics and medium-term outcomes of HF in Ghana by outlining the epidemiological and clinical characteristics of patients with HF in Ghana, identifying the underlying causes, evaluating the medium-term outcomes of HF in Ghana, and identifying the factors that predict hospitalization and mortality in patients with HF in Ghana. This study also aims to build capacity in the care of patients with HF and form the basis for a national registry for HF in Ghana.

## Methods

### Overview

**Figure 1:**
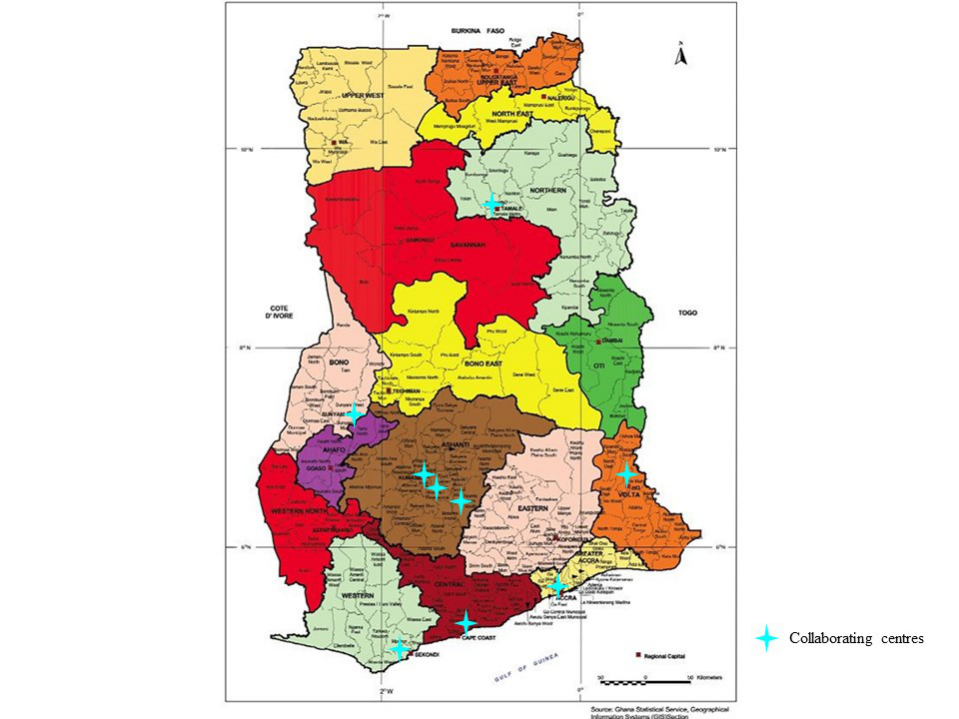
Map of Ghana showing the location of collaborating centers. Source: Ghana Statistical Service, 2020.

An NNHFMT consisting of physicians, nurses, and researchers from 9 collaborating hospitals in Ghana was constituted to achieve the study objectives, with each institutional HFMT led by a cardiologist, 2 supporting cardiologists or physicians, and 2 nurses. The HFMTs will establish HF clinics and integrate them into the routine services of their hospitals; recruit patients with HF and manage them per guidelines and algorithm; and create awareness and train other personnel in the 9 centers on the diagnosis and management of HF. These institutions include 5 teaching hospitals, 3 regional hospitals, and 1 municipal hospital. They include the Korle-Bu Teaching Hospital (KBTH), Komfo Anokye Teaching Hospital (KATH), Tamale Teaching Hospital (TTH), Ho Teaching Hospital (HTH), Cape Coast Teaching Hospital (CCTH), Bono Regional Hospital, Presbyterian Hospital-Agogo, Kumasi South Hospital, and the Effia Nkwanta Regional Hospital (Figure 1) [18].

### Study Design

**Figure 2:**
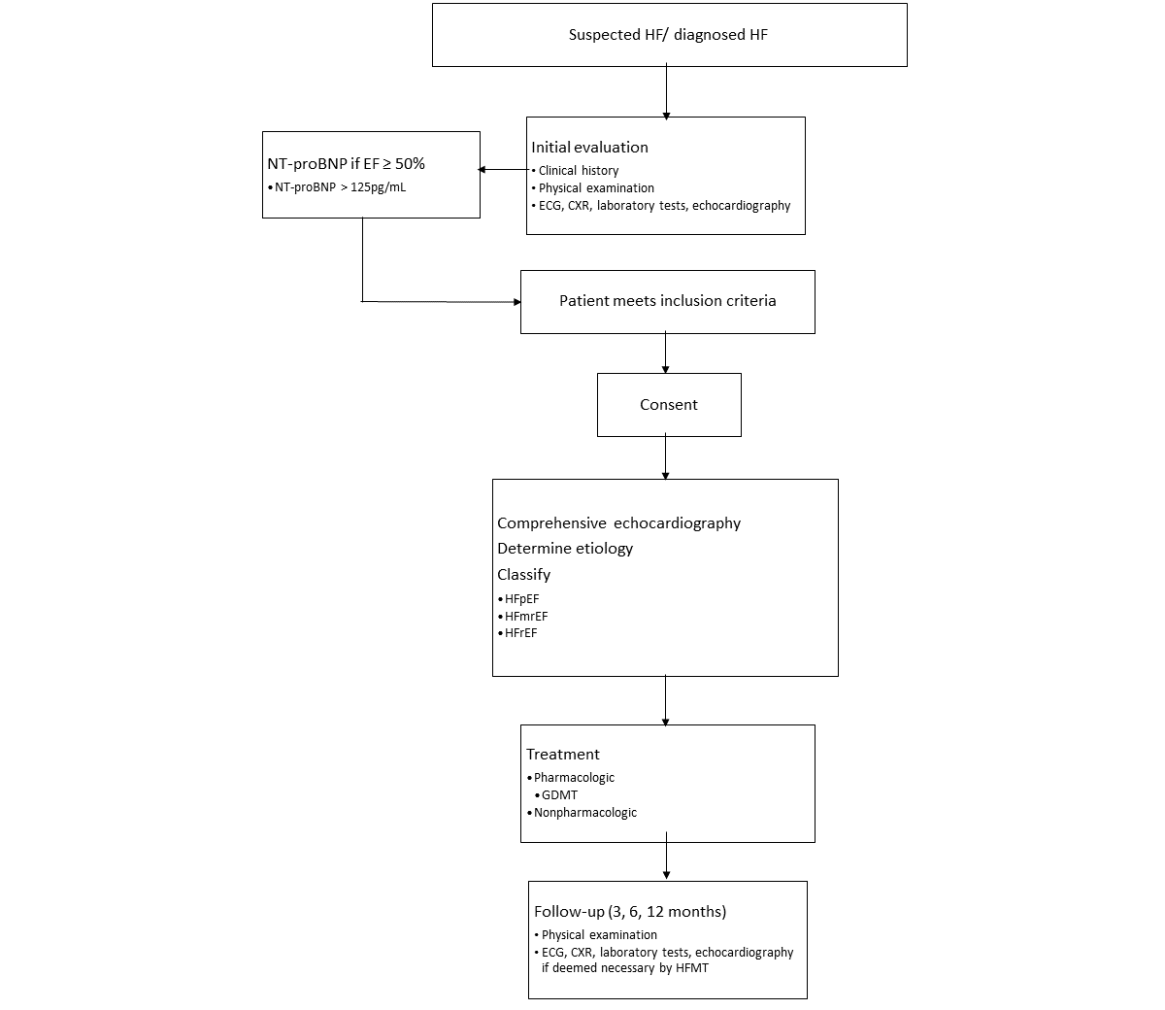
Chart outlining study design.

This is a prospective, multicenter, and multilevel observational study of patients with HF. Patients presenting with HF will be recruited and evaluated according to a standardized protocol. Guideline-directed treatment for HF will then be prescribed after the diagnosis has been confirmed. The study will conduct a serum N-terminal pro-brain natriuretic peptide (NT-proBNP) and transthoracic echocardiogram for all participants for free, while the cost of treatment and other investigations will be borne by study participants as in routine care. Patients will be followed up prospectively for 6 months to determine the medium-term outcomes of interventions (Figure 2).

Patients will be recruited through the collaborating institutions’ emergency rooms, admission wards, and established HF clinics from January to December 2023. The HFMTs of the 2 leading teaching hospitals in Ghana, KBTH and KATH, will be trained using a facilitators’ training manual, which will be developed from current international HF guidelines. KBTH and KATH will then provide mentorship and training to the other collaborating hospitals. The HFMT of KBTH will train members of the HFMTs from the TTH, HTH, and Effia-Nkwanta Regional Hospital, while the HFMT of KATH will also train the HFMTs of CCTH, Bono Regional Hospital, Presbyterian Hospital-Agogo, and Kumasi South Hospital. The study will begin in the KBTH and KATH in January 2023, while the other 7 sites will begin recruitment in July 2023, and all areas will end enrollment in December 2023.

### Study Population

Study participants will include patients aged 13 years or older who present with HF in the collaborating hospitals and consent to participate in the study. Patients with a life expectancy less than the expected duration of the registry due to non-HF comorbidities will be excluded.

Based on our estimation of a sample size of 5000 participants, we would be able to determine the mortality and hospitalization rate at 6 months with a 95% CI and a precision of ±1%. Each participating center will recruit 556 participants.

### Recruitment of Study Participants

Participants will be recruited through the various departments or units of the collaborating institutions. All patients diagnosed with HF or suspected of having HF will be referred to the HFMTs for evaluation and enrollment. A total of 2 sensitization workshops will be organized at collaborating institutions during the study period: 1 before participants’ enrollment and 1 midway through participant recruitment.

### Data Collection

Data collection comprises administering questionnaires, reviewing medical records, physical examinations, imaging investigations, including chest x-rays and echocardiography, electrocardiography, and laboratory tests. The methods for data collection in this study are identical in all locations, following standardized operating manuals and tools.

### Diagnosis of HF

**Figure 3:**
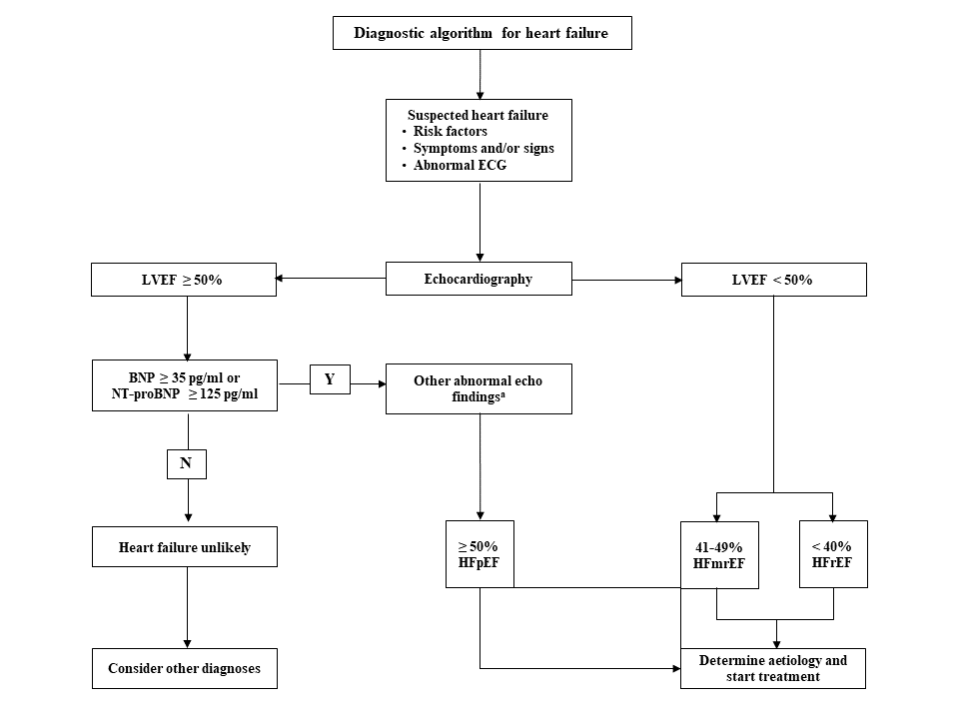
Modified diagnostic algorithm for heart failure (HF). BNP: brain natriuretic peptide; ECG: electrocardiogram; HFmrEF: heart failure with mildly reduced ejection fraction; HFpEF: heart failure with preserved ejection fraction; HFrEF: heart failure with reduced ejection fraction; LVEF: left ventricular ejection fraction.

The diagnosis of HF will be made using a modified diagnostic algorithm adopted from the 2021 European Society of Cardiology Guidelines to diagnose and treat acute and chronic HF (Figure 3) [19]. The diagnosis of HF will be made based on the presence of typical symptoms of HF and objective evidence of cardiac dysfunction and categorized into three phenotypes: (1) heart failure with reduced ejection fraction (HFrEF), (2) heart failure with mildly reduced ejection fraction (HFmrEF), and (3) heart failure with preserved ejection fraction (HFpEF) based on the left ventricular ejection fraction (LVEF) [19].

All patients with typical symptoms and specific signs of HF and LVEF≤40% will be categorized as HFrEF, while patients with LVEF of 41%-49 % will be categorized as HFmrEF.

HFpEF will be diagnosed in patients presenting with typical symptoms and specific signs of HF and LVEF≥50%, the presence of elevated natriuretic peptides (NT-proBNP≥125 pg/mL), and objective evidence of cardiac structural and functional abnormalities consistent with the presence of left ventricular (LV) diastolic dysfunction or raised LV filling pressures. Objective evidence of structural or functional abnormalities includes the following:

- LV mass index ≥95 g/m^2^ (female), ≥115 g/m^2^ (male), and a relative wall thickness >0.42.
- Left atrial volume index >34 mL/m^2^ in sinus rhythm (SR) and the presence of atrial fibrillation (AF) left atrial volume >40 mL/m^2^.
- E/e’ ratio at rest >9.
- NT-proBNP >125 (SR) or >365 (AF) pg/mL OR BNP >35 (SR) or >105 (AF) pg/mL.
- Pulmonary artery systolic pressure >35 mm Hg or tricuspid regurgitant velocity at rest >2.8 m/second.

### Etiology of HF

The etiology of HF will be determined based on the history, physical examination, laboratory, electrocardiographic, echocardiographic findings, and other imaging modalities (Table 1).

**Table 1:** Heart failure (HF) etiology and clinical characteristics.

Etiology of HF	Clinical characteristics	Specific investigations
Hypertension [20-22]	Persistent elevated systolic BP^a^ ≥140 mm Hg and diastolic BP ≥90 mm Hg Presence of HMOD^b^ Current or previous use of antihypertensive medications	24-hour ambulatory BP Plasma metanephrines and renal artery imaging Serum renin and aldosterone TTE^c^
Coronary artery disease [23]	HF and ACS^d^ A pre-existing history of CCS^e^ Features suggestive of significant CAD^f^ on coronary angiography or other imaging	Invasive coronary angiography CT^g^ coronary angiography Imaging stress tests (echo, nuclear, and CMR^h^)
Valvular heart disease [24]	Primary valve disease, for example, aortic stenosis Secondary valve disease, for example, functional regurgitation Congenital valve disease, for example, bicuspid aortic valve and mitral valve prolapse	TTE/ TEE^i^/ stress echo CT/ CMR
Rheumatic heart disease [25,26]	Primary valve disease, for example, mitral stenosis and mitral regurgitation Atrial fibrillation	TTE/ TEE/ stress echo
Dilated cardiomyopathy [27,28]	Unexplained dilated cardiac chambers with increased left ventricular mass index	CMR, genetic testing Trace elements, toxicology, LFTs^j^
Arrhythmia-induced cardiomyopathy [29]	Mean heart rate above 100 beats per minute Atrial fibrillation Premature ventricular contractions burden equal to or greater than 10% No other cause of LV dysfunction identified	Ambulatory ECG recording Electrophysiology study, if indicated
Congenital heart disease	History of congenital heart disease Incidental diagnosis of congenital heart disease during investigation for HF	TTE/ TEE CMR
Other etiologies of HF [19]	Clinical features diagnostic of restrictive cardiomyopathy, arrhythmogenic cardiomyopathy, peripartum cardiomyopathy, endomyocardial fibrosis, cor pulmonale, infiltrative cardiomyopathy, pericardial disease, LV noncompaction cardiomyopathy, and toxin-induced cardiomyopathy.	Serum electrophoresis and serum free light chains. Echo, CMR, CT-PET^k^, endomyocardial biopsy, Serum angiotensin-converting enzyme, fluorodeoxyglucose-PET, and chest CT Right and left heart catheterization

^a^BP: blood pressure.

^b^HMOD: hypertension-mediated organ damage.

^c^TTE: transthoracic echocardiogram.

^d^ACS: acute coronary syndrome.

^e^CCS: chronic coronary syndrome.

^f^CAD: coronary artery disease.

^g^CT: computed tomography.

^h^CMR: cardiovascular magnetic resonance.

^i^TEE: transesophageal echocardiogram.

^j^LFT: liver function test.

^k^PET: positron emission tomography.

### Treatment of HF

**Figure 4:**
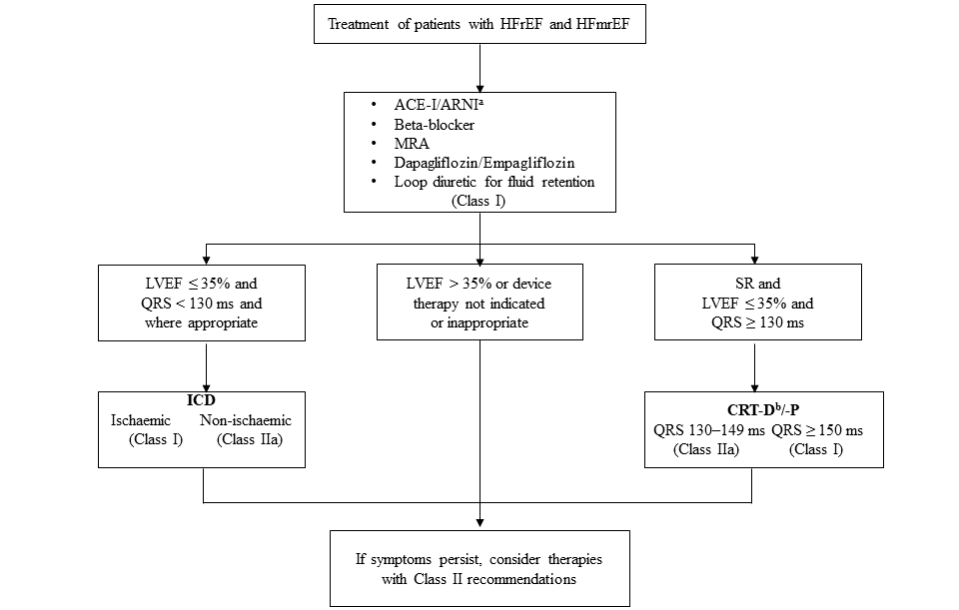
Modified treatment algorithm for treating HFrEF and HFmrEF. a: a replacement for ACE-I/ARB (angiotensin receptor blocker); ACE-I: angiotensin-converting enzyme inhibitor; ARNI: angiotensin receptor-neprilysin inhibitor; CRT-D: cardiac resynchronisation therapy with a defibrillator; CRT-P: cardiac resynchronisation therapy pacemaker; HFmrEF: heart failure with mildly reduced ejection fraction; HFrEF: heart failure with reduced ejection fraction; ICD: implantable cardioverter-defibrillator; MRA: mineralocorticoid receptor antagonist; ms: milliseconds; SR: sinus rhythm.

A modified treatment algorithm adopted from the 2021 European Society of Cardiology Guidelines for treating acute and chronic HF (Figure 4) will be used in the treatment of patients [19].

### Data Handling and Analysis

The KoboCollect toolbox (v2022.4.4; [30]) will be used to capture data and upload it onto a cloud database that is only accessible to the principal investigator and the data manager. Collated data will be exported into SPSS (*package 2016*; SPSS Inc) for statistical analysis. Tables, bar charts, and pie charts will be used to present the data. For continuous variables, the central tendency and spread measures will be calculated using the mean (SD), and IQR. Categorical variables will be reported as numbers and percentages. Multivariable regression models will be used explore the relationships between variables and rehospitalization and death. We will perform a Kaplan-Meier analysis to estimate the survival and death rate of patients with HF. The Cox regression analysis will determine the relationship between the risk of death in an individual and selected variables and the significance of these variables. Missing values will be handled based on the type and frequency of missing values. A P value <.05 will be considered statistically significant.

### Timelines

The table of timelines (Table 2) below summarizes the key activities of the study from the start to the end of the study.

**Table 2:** Table of timelines.

	2022	2023													2024	
	December	January	February	March	April	May	June	July	August	September	October	November	December	January-June		June
Training for KBTH and KATH	✓						✓									
Training for other sites								✓			✓					
Sensitization workshops for KBTH and KATH	✓					✓										
Sensitization workshops for other sites								✓			✓					
Enrollment and data collection		✓	✓	✓	✓	✓	✓	✓	✓	✓	✓	✓	✓			
Establishment of HF clinics		✓						✓								
Follow-up				✓	✓	✓	✓	✓	✓	✓	✓	✓	✓	✓		
Preliminary data analysis and reporting										✓						
End of enrollment														✓		
Data analysis									✓	✓	✓	✓	✓	✓		
End of study																✓

### Ethical Considerations

Ethical approval has been obtained from the Ethical and Institutional Review Boards of Korle-Bu Teaching Hospital (STC/IRB/000150/2022), Cape Coast Teaching Hospital (CCTHERC/EC/2023/019), Tamale Teaching Hospital (TTH/R&D/SR/229), Ho Teaching Hospital (HTH-REC (30) FC_2023), and Komfo Anokye Teaching Hospital (KATH IRB/AP/166/22). All participants will be informed about the study, its objectives, and the data collection methods. Consent will be obtained from participants who agree to be part of the study and are assured of strict confidentiality and anonymity. Participants will also be informed that participation in the study is entirely voluntary, and all the services they receive at the clinic will continue as usual whether they decide to participate or not. A unique study number will be assigned to each participant, and the collected data will be deidentified. Only the assigned unique number will be used on study documents related to participants. Participants will also be informed that they can withdraw from the study at any time they choose without consequences.

## Results

This intervention will generate the necessary information on the etiology of HF, clinical presentations, the diagnostic yield of various tools, and management outcomes. In addition, it will build the necessary capacity and support for HF management in Ghana. As of July 30, 2023, the training of the various health workers in all 9 hospitals has been completed. The KATH and the KBTH acted as supervisory sites and supported the training at 4 and 3 sites, respectively. Preliminary analyses will be conducted by the end of the second quarter of 2024, and results are expected to be publicly available by the middle of 2024.

The test-run of the research and registry instruments and modifications have been completed. Medical equipment (echocardiogram machines and NT-proBNP devices) has been calibrated and distributed to all sites.

## Discussion

### Overview

HF is one of the leading causes of hospital admissions in developing countries and is predicted to experience the most rapid growth worldwide [2,7,31,32]. This prospective study will answer many clinical questions about HF in Ghana. First, this study will address a notable data scarcity in HF care and research in Ghana by establishing a national HF registry, thus creating a vehicle for the accrual of large, comprehensive, and contemporary data encompassing the sociodemographic, clinical profiles, causes of HF, management of HF, and determinants of outcomes such as mortality and hospitalizations of patients with HF in Ghana. In addition, a national HF registry will also provide a good opportunity to evaluate adherence to current guidelines and response to treatment among patients with HF in Ghana.

Clinical registries play a crucial role in gathering real-world data, essential for developing evidence for best clinical practice, measuring outcomes, providing feedback to clinicians, and enhancing the quality of care [1,33]. HF registries, like the Swedish Heart Failure Registry, have played a significant role in advancing knowledge and improving the management of HF. Established in 2000 and implemented nationwide in Sweden by 2003, this registry has yielded valuable research outcomes that have led to notable improvements in the understanding and care of patients with HF and under-treatment detection [1]. The NAtional TUnisian REgistry of Heart Failure (NATURE-HF) contributed valuable data that have the potential to enhance the treatment and overall prognosis of individuals with HF in North Africa [34]. Valuable data were also derived from the Abeokuta Heart Failure Clinical Registry of patients presenting with acute HF in Abeokuta, Nigeria, including acute HF presenting at a relatively younger age, commoner in men, and associated with severe symptoms [35].

While the acquisition of realistic data will address the data gap, this pragmatic study will provide capacity building for the management of HF by enhancing the skills and knowledge of health care providers in diagnosing and treating HF and making diagnostic equipment, including echocardiography, electrocardiograms, and point-of-care NT-proBNP devices, available to participating hospitals. Furthermore, participating institutions’ HFMTs and HF clinics will broaden the prospect for specialist HF care and long-term follow-up in their regions.

Although numerous HF guidelines are available to aid in managing patients with HF, their generalizability presents variable challenges, as these guidelines may not be appropriate for managing HF in countries with limited health care resources [19]. Therefore, we anticipate that this study will serve as a significant milestone in establishing a standardized approach to managing HF in Ghana and the wider SSA region.

The study will address a notable void within Ghana’s ever-evolving HF care and research domain. The study will generate novel and indispensable data that will improve HF care, serve as a foundation for teaching, develop locally tailored HF guidelines, and establish HF research programs.

### Strengths and Limitations of This Study

This pragmatic, prospective, multicenter study will generate the most extensive contemporary data on HF in Ghana. The study will also enhance the knowledge and skills of health personnel in diagnosing and managing HF. The project is also designed to establish HF clinics and provide diagnostic services as part of routine health care services in participating hospitals. This study will describe associations rather than establish causality owing to its observational design.

## Abbreviations

AF: atrial fibrillation
CCTH: Cape Coast Teaching Hospital
HF: heart failure
HFmrEF: heart failure with mildly reduced ejection fraction
HFMT: heart failure management team
HFpEF: heart failure with preserved ejection fraction
HFrEF: heart failure with reduced ejection fraction
HTH: Ho Teaching Hospital
KATH: Komfo Anokye Teaching Hospital
KBTH: Korle-Bu Teaching Hospital
LV: left ventricular
LVEF: left ventricular ejection fraction
NATURE-HF: NAtional TUnisian REgistry of Heart Failure
NNHFMT: national network of heart failure management teams
NT-proBNP: N-terminal pro-brain natriuretic peptide
SR: sinus rhythm
SSA: Sub-Saharan Africa
TTH: Tamale Teaching Hospital

## Data Availability

The data sets generated and analyzed during this study will be available from the corresponding author on reasonable request following completion of the study and publication of the findings of the study.

## References

[1] Savarese G, Becher PM, Lund LH, Seferovic P, Rosano GMC, Coats AJS (2023). Global burden of heart failure: a comprehensive and updated review of epidemiology. Cardiovasc Res.

[2] Gtif I, Bouzid F, Charfeddine S, Abid L, Kharrat N (2021). Heart failure disease: an African perspective. Arch Cardiovasc Dis.

[3] Ogah OS, Adebiyi A, Sliwa K, Rescigno G, Firstenberg MS (2019). Heart failure in Sub-Saharan Africa. Topics in Heart Failure Management.

[4] Agbor VN, Essouma M, Ntusi NAB, Nyaga UF, Bigna JJ, Noubiap JJ (2018). Heart failure in sub-Saharan Africa: a contemporaneous systematic review and meta-analysis. Int J Cardiol.

[5] Adidja NM, Agbor VN, Aminde JA, Ngwasiri CA, Ngu KB, Aminde LN (2018). Non-adherence to antihypertensive pharmacotherapy in Buea, Cameroon: a cross-sectional community-based study. BMC Cardiovasc Disord.

[6] Mensah GA, Roth GA, Sampson UKA, Moran AE, Feigin VL, Forouzanfar MH, Naghavi M, Murray CJL (2015). Mortality from cardiovascular diseases in sub-Saharan Africa, 1990-2013: a systematic analysis of data from the Global Burden of Disease Study 2013. Cardiovasc J Afr.

[7] Appiah LT, Sarfo FS, Agyemang C, Tweneboah HO, Appiah NABA, Bedu-Addo G, Opare-Sem O (2017). Current trends in admissions and outcomes of cardiac diseases in Ghana. Clin Cardiol.

[8] Bonsu KO, Owusu IK, Buabeng KO, Reidpath DD, Kadirvelu A (2017). Clinical characteristics and prognosis of patients admitted for heart failure: a 5-year retrospective study of African patients. Int J Cardiol.

[9] Bragazzi NL, Zhong W, Shu J, Much AA, Lotan D, Grupper A, Younis A, Dai H (2021). Burden of heart failure and underlying causes in 195 countries and territories from 1990 to 2017. Eur J Prev Cardiol.

[10] Owusu AY, Kushitor SB, Ofosu AA, Kushitor MK, Ayi A, Awoonor-Williams JK (2021). Institutional mortality rate and cause of death at health facilities in Ghana between 2014 and 2018. PLoS One.

[11] Karaye KM, Dokainish H, ElSayed A, Mondo C, Damasceno A, Sliwa K, Balasubramanian K, Grinvalds A, Yusuf S (2021). Clinical profiles and outcomes of heart failure in five African countries: results from INTER-CHF study. Glob Heart.

[12] Owusu IK, Adu-Boakye Y (2013). Prevalence and aetiology of heart failure in patients seen at a teaching hospital in Ghana. J Cardiovasc Dis Diagn.

[13] Yuyun MF, Sliwa K, Kengne AP, Mocumbi AO, Bukhman G (2020). Cardiovascular diseases in sub-Saharan Africa compared to high-income countries: an epidemiological perspective. Glob Heart.

[14] Njoroge JN, Teerlink JR (2021). Pathophysiology and therapeutic approaches to acute decompensated heart failure. Circ Res.

[15] Dokainish H, Teo K, Zhu J, Roy A, AlHabib KF, ElSayed A, Palileo-Villaneuva L, Lopez-Jaramillo P, Karaye K, Yusoff K, Orlandini A, Sliwa K, Mondo C, Lanas F, Prabhakaran D, Badr A, Elmaghawry M, Damasceno A, Tibazarwa K, Belley-Cote E, Balasubramanian K, Yacoub MH, Huffman MD, Harkness K, Grinvalds A, McKelvie R, Yusuf S (2016). Heart failure in Africa, Asia, the Middle East and South America: the INTER-CHF study. Int J Cardiol.

[16] Morton G, Masters J, Cowburn PJ (2018). Multidisciplinary team approach to heart failure management. Heart.

[17] Grady KL, Dracup K, Kennedy G, Moser DK, Piano M, Stevenson LW, Young JB (2000). Team management of patients with heart failure: a statement for healthcare professionals from the Cardiovascular Nursing Council of the American Heart Association. Circulation.

[18] (2019). Know the 16 regional capitals of Ghana. Daily Graphic Online.

[19] McDonagh TA, Metra M, Adamo M, Gardner RS, Baumbach A, Böhm M, Burri H, Butler J, Čelutkienė J, Chioncel O, Cleland JGF, Coats AJS, Crespo-Leiro MG, Farmakis D, Gilard M, Heymans S, Hoes AW, Jaarsma T, Jankowska EA, Lainscak M, Lam CSP, Lyon AR, McMurray JJV, Mebazaa A, Mindham R, Muneretto C, Piepoli MF, Price S, Rosano GMC, Ruschitzka F, Skibelund AK (2021). 2021 ESC guidelines for the diagnosis and treatment of acute and chronic heart failure. Eur Heart J.

[20] Owusu IK, Adu-Boakye Y, Tetteh LA (2014). Hypertensive heart failure in Kumasi, Ghana. Open Sci J Clin Med.

[21] Mancia G, Dominiczak A (2020). The new international society of hypertension guidelines on hypertension. J Hypertens.

[22] Williams B, Mancia G, Spiering W, Rosei EA, Azizi M, Burnier M, Clement DL, Coca A, de Simone G, Dominiczak A, Kahan T, Mahfoud F, Redon J, Ruilope L, Zanchetti A, Kerins M, Kjeldsen SE, Kreutz R, Laurent S, Lip GYH, McManus R, Narkiewicz K, Ruschitzka F, Schmieder RE, Shlyakhto E, Tsioufis C, Aboyans V, Desormais I (2018). 2018 ESC/ESH guidelines for the management of arterial hypertension. Eur Heart J.

[23] Knuuti J, Wijns W, Saraste A, Capodanno D, Barbato E, Funck-Brentano C, Prescott E, Storey RF, Deaton C, Cuisset T, Agewall S, Dickstein K, Edvardsen T, Escaned J, Gersh BJ, Svitil P, Gilard M, Hasdai D, Hatala R, Mahfoud F, Masip J, Muneretto C, Valgimigli M, Achenbach S, Bax JJ (2020). 2019 ESC guidelines for the diagnosis and management of chronic coronary syndromes: the Task Force for the diagnosis and management of chronic coronary syndromes of the European Society of Cardiology (ESC). Eur Heart J.

[24] Vahanian A, Beyersdorf F, Praz F, Milojevic M, Baldus S, Bauersachs J, Capodanno D, Conradi L, De Bonis M, De Paulis R, Delgado V, Freemantle N, Gilard M, Haugaa KH, Jeppsson A, Jüni P, Pierard L, Prendergast BD, Sádaba JR, Tribouilloy C, Wojakowski W (2022). 2021 ESC/EACTS guidelines for the management of valvular heart disease: developed by the Task Force for the management of valvular heart disease of the European Society of Cardiology (ESC) and the European Association for Cardio-Thoracic Surgery (EACTS). Eur Heart J.

[25] Reményi B, Wilson N, Steer A, Ferreira B, Kado J, Kumar K, Lawrenson J, Maguire G, Marijon E, Mirabel M, Mocumbi AO, Mota C, Paar J, Saxena A, Scheel J, Stirling J, Viali S, Balekundri VI, Wheaton G, Zühlke L, Carapetis J (2012). World Heart Federation criteria for echocardiographic diagnosis of rheumatic heart disease—an evidence-based guideline. Nat Rev Cardiol.

[26] Nunes MCP, Sable C, Nascimento BR, de Lima EM, da Silva JLP, Diamantino AC, Oliveira KKB, Okello E, Aliku T, Lwabi P, Colosimo EA, Ribeiro ALP, Beaton AZ (2019). Simplified echocardiography screening criteria for diagnosing and predicting progression of latent rheumatic heart disease. Circ Cardiovasc Imaging.

[27] Japp AG, Gulati A, Cook SA, Cowie MR, Prasad SK (2016). The diagnosis and evaluation of dilated cardiomyopathy. J Am Coll Cardiol.

[28] Pinto YM, Elliott PM, Arbustini E, Adler Y, Anastasakis A, Böhm M, Duboc D, Gimeno J, de Groote P, Imazio M, Heymans S, Klingel K, Komajda M, Limongelli G, Linhart A, Mogensen J, Moon J, Pieper PG, Seferovic PM, Schueler S, Zamorano JL, Caforio ALP, Charron P (2016). Proposal for a revised definition of dilated cardiomyopathy, hypokinetic non-dilated cardiomyopathy, and its implications for clinical practice: a position statement of the ESC working group on myocardial and pericardial diseases. Eur Heart J.

[29] Huizar JF, Ellenbogen KA, Tan AY, Kaszala K (2019). Arrhythmia-induced cardiomyopathy: JACC State-of-the-Art review. J Am Coll Cardiol.

[30] KoboToolBox.

[31] Kraus S, Ogunbanjo G, Sliwa K, Ntusi NAB (2016). Heart failure in sub-Saharan Africa: a clinical approach. S Afr Med J.

[32] Ogah OS, Stewart S, Onwujekwe OE, Falase AO, Adebayo SO, Olunuga T, Sliwa K (2014). Economic burden of heart failure: investigating outpatient and inpatient costs in Abeokuta, Southwest Nigeria. PLoS One.

[33] Meltzer SN, Weintraub WS (2020). The role of national registries in improving quality of care and outcomes for cardiovascular disease. Methodist Debakey Cardiovasc J.

[34] Abid L, Kammoun I, Ben Halima M, Charfeddine S, Ben Slima H, Drissa M, Mzoughi K, Mbarek D, Riahi L, Antit S, Ben Halima A, Ouechtati W, Allouche E, Mechri M, Yousfi C, Khorchani A, Abid O, Sammoud K, Ezzaouia K, Gtif I, Ouali S, Triki F, Hamdi S, Boudiche S, Chebbi M, Hentati M, Farah A, Triki H, Ghardallou H, Raddaoui H, Zayed S, Azaiez F, Omri F, Zouari A, Ben Ali Z, Najjar A, Thabet H, Chaker M, Mohamed S, Chouaieb M, Ben Jemaa A, Tangour H, Kammoun Y, Bouhlel M, Azaiez S, Letaief R, Maskhi S, Amri A, Naanaa H, Othmani R, Chahbani I, Zargouni H, Abid S, Ayari M, Ben Ameur I, Gasmi A, Ben Halima N, Haouala H, Boughzela E, Zakhama L, Ben Youssef S, Nasraoui W, Boujnah MR, Barakett N, Kraiem S, Drissa H, Ben Khalfallah A, Gamra H, Kachboura S, Bezdah L, Baccar H, Milouchi S, Sdiri W, Ben Omrane S, Abdesselem S, Kanoun A, Hezbri K, Zannad F, Mebazaa A, Kammoun S, Mourali MS, Addad F (2021). Design and rationale of the national Tunisian registry of heart failure (NATURE-HF): protocol for a multicenter registry study. JMIR Res Protoc.

[35] Ogah OS, Stewart S, Falase AO, Akinyemi JO, Adegbite GD, Alabi AA, Ajani AA, Adesina JO, Durodola A, Sliwa K (2014). Contemporary profile of acute heart failure in Southern Nigeria: data from the Abeokuta heart failure clinical registry. JACC Heart Fail.

